# Everybody's Page

**Published:** 1889-10-26

**Authors:** 


### Everybody's Page.

A damp subsoil predisposes to consumption, probably because such a soil tends to make those living on it catch cold easily.

Dr. Robert Lee thinks that inhaling a vapour formed of one per cent. of carbolic acid in boiling water is the best remedy for the laryngeal symptoms of whooping cough.

A very simple method of inducing sleep in cases of persistent insomnia, and one that has succeeded where many drugs have failed, is simply to administer a moderate amount of warm liquid food before the patient goes to bed. This diverts the blood from the brain to the abdominal organs, and takes away the cerebral excitement that precludes sleep.

The annual income of the voluntary rate-aided and other medical charities of London is about £1,196,471, of which about £485,502 comes from rates, £160,000 from endowments, £100,000 from legacies, £50,000 from Hospital Saturday and Sunday Funds, £50,000 from patients' payments, and £350,000 from subscriptions, donations, and the proceeds of bazaars and other entertainments.

There grows in Eastern Arabia a plant which produces effects similar to those of laughing-gas. When the seeds of it are ground and administered to anyone, he begins to behave in a peculiar manner. He laughs loudly, sings, dances, and generally behaves in an insane fashion. After about an hour the excitement ends, and the exhausted patient falls into a deep sleep, from which he awakens without any recollection of his past eccentricities.

Ostrich farming is now being taken up in the Australian colonies, and the feathers obtained are said to be as good as those of the African birds.

"Oil on the troubled waters" is not a mere phrase, but the statement of a practical fact, and some sailors are taking cans of oil, to be used to prevent the buffeting of the waves in storms.

Very young children are not sensitive to pain to any great extent. Dr. Geuger calculates that this sensibility is seldom clearly shown in less than four or five weeks after birth, and before that time infants do not shed tears.

There are many chronic affections of the heart which are due to rheumatism. The pain is intermittent, and is greatly affected by changes in the weather. Sometimes rheumatic sore throat appears in people who have never suffered from rheumatism, but, although no history of the disease may be traceable, no treatment is effective that does not take the diathesis into account.

Mr. Shirley Murphy related an anecdote before the Epidemiological Society some time ago which threw a pleasing light on many cases of infection through milk. On a farm where some cows were suffering from disease of the udder, one of the dairymen remarked that "they could not drink the milk themselves, so they sent it to London, but they hoped the poor people there would not suffer." And doubtless that dairyman is conscientiously shocked when he hears of a murder.

